# Monocular ictal nystagmus in a dog: potentially a newly recognized focal seizure phenotype

**DOI:** 10.1093/jvimsj/aalaf078

**Published:** 2026-01-27

**Authors:** João Miguel De Frias, Elsa Lyon, Albert Aguilera-Padros, Aran Nagendran

**Affiliations:** The Royal (Dick) School of Veterinary Studies Hospital for Small Animals, University of Edinburgh, Edinburgh EH25 9RG, United Kingdom; Elyope, 94100 Saint-Maur-des-Fossés, France; The Royal (Dick) School of Veterinary Studies Hospital for Small Animals, University of Edinburgh, Edinburgh EH25 9RG, United Kingdom; The Royal (Dick) School of Veterinary Studies Hospital for Small Animals, University of Edinburgh, Edinburgh EH25 9RG, United Kingdom

**Keywords:** canine, focal seizure, saccadic eye movement, vestibular epilepsy

## Abstract

A 3-year-old, male neutered toy Chinese crested powderpuff dog was presented with an acute onset obtundation that progressed to status epilepticus. On presentation, neurological examination was localized to a right forebrain lesion. Bizarre episodes, consisting of disconjugate nystagmus of the left eye, medial strabismus of the right eye with convergent-retraction movements in both eyes, were recorded. Head magnetic resonance imaging revealed intra-axial multifocal lesions affecting the right fronto-temporal cortices and dorsal paramedian thalamus. Cerebrospinal fluid analysis revealed a marked mononuclear pleocytosis. Electroencephalographic recordings revealed recurrent medium-amplitude interictal isolated spikes, and suspected epileptic spikes alongside with eye movement that were mainly visible in the right hemisphere. The presumptive diagnosis was meningoencephalitis of unknown origin. Despite treatment, the dog died. This is a report of monocular nystagmus with a presumptive epileptic origin in veterinary medicine, a rare clinical sign in human patients.

## Case report

A 3-year-old, 7.95 kilograms, male castrated toy Chinese crested powderpuff dog was presented with a 24-hour history of an acute onset of obtundation that progressed to a continuous generalized tonic–clonic seizure. On arrival at the general practitioner, the dog was actively seizing with a generalized tonic–clonic phenotype for approximately 10 minutes (status epilepticus). Convulsions resolved after administration of diazepam (1 mg/kg IV), and, when recovered from seizure, a loading dose of levetiracetam (60 mg/kg PO) was administered. Hematology and serum biochemistry were performed, and no abnormalities were detected. No access to toxins was reported. The dog was subsequently transferred to the neurology and neurosurgery service at the referral institution after 4 hours, with no further obvious seizure activity. On arrival, the dog had a normal cardiopulmonary auscultation, with a normal heart rate (130 beats per minute) and respiratory rate (40 breaths per minute). He was normothermic (38° C), well hydrated and with a normal body perfusion (Doppler non-invasive arterial blood pressure was 130 mmHg). Blood gases, electrolytes, glucose and ammonia were within normal limits. Neurological examination abnormalities included a moderate to marked obtundation, head turn to the left, moderate generalized proprioceptive ataxia, delayed postural reactions on the left thoracic and pelvic limb and normal on the right side, an absent menace response on the left eye and reduced left nasal septal mucosal sensation (Video 1). On presentation, no pathological positional or static nystagmus were detected. The neuroanatomical localization was consistent with a right forebrain lesion.

Whilst hospitalized, the dog developed further seizure activity. A tremor of the left ear was initially detected and there was a disconjugate abnormal nystagmus of the left eye (Video 2). The left eye was observed to have a combination of jerk movements mainly horizontally with a fast phase towards the left side. Intermittent vertical nystagmus was also perceived. Both eyes exhibited a retraction of eye globe in a spasmodic movement leading to a partial closure of eyelids. There was a convergent movement of the right eye in a synchronous fashion. While the left eye monocular nystagmus was observed, there was a persistent medial (convergent) strabismus of the right eye. About three similar episodes were noticed which lasted less than a minute. Whilst ocular episodes were occurring, there was an exacerbation of the previously noticed head turn to the left and during the episodes the dog was markedly obtunded with no response to his surrounding environment. These episodes were followed by an asymmetrical non-generalized tonic–clonic seizure, with twitching more evident in left than the right side of the face (ears and jaw) and autonomic signs (hypersalivation) (Video 3). All seizure activity noticed lasted around 15 seconds. A bolus of diazepam (Ziapam, Domes Pharma, UK; 0.5 mg/kg IV) was administered during asymmetrical non-generalized tonic–clonic seizure which stopped activity. This was followed by administration of levetiracetam (Keppra, UCB Pharma, Belgium; 30 mg/kg IV q8h). Overnight, only a short and subtle left sided monocular nystagmus was reported, but no further interventions were performed. The dog was also perceived to be nauseous with excessive drooling and maropitant (Prevomax, Dechra, UK; 1 mg/kg IV) was administered.

**Figure 1:**
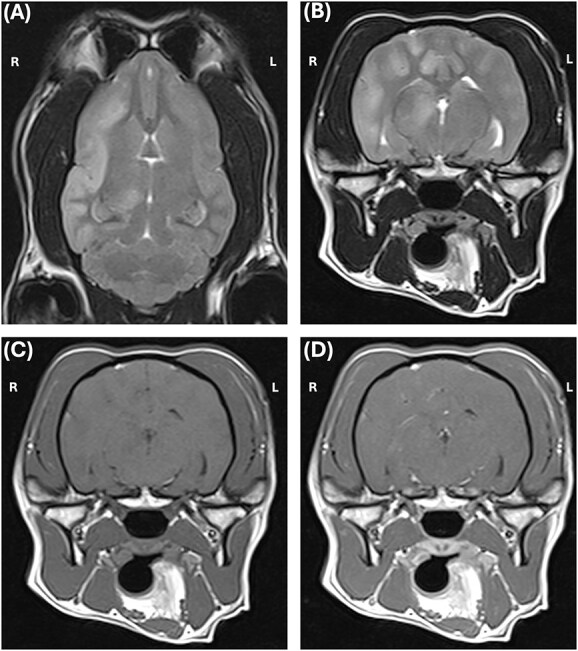
Magnetic resonance imaging of the head the dog with a T2-weighted (T2W) in dorsal (A) and transverse (B) planes and T1-weighted (T1W) pre (C) and post-contrast (T1W + C) (D). There are intra-axial multifocal lesions in the right fronto-temporal regions and dorsal paramedian thalamus that were mainly affecting diffusely the gray matter; T2W hyperintense, T2W, T1W isointense and with “patchy” parenchymal and mild leptomeningeal enhancement in T1W + C.

**Figure 2:**
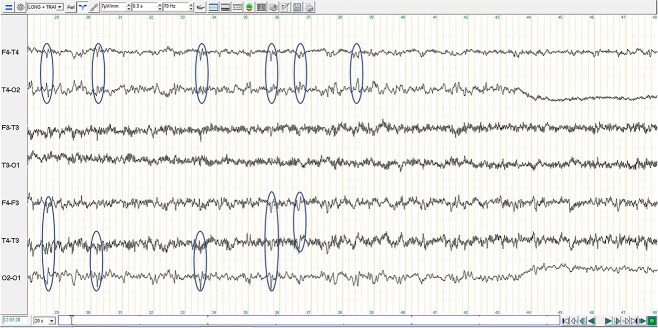
Extract of 20 seconds of the electroencephalographic examination in longitudinal (F2–T4, T4–O2, F3–T3, T3–O1) and transverse (F4–F3, T4–T3, O2–O1) bipolar montage for this case. Isolated and recurrent spikes are observed (circled in the figure) on the channels covering the right hemisphere in longitudinal montage (F4–T4 and T4–O2) and on all the channels in transverse montage (F4–T3, T4–T3 and O2–O1).

**Figure 3:**
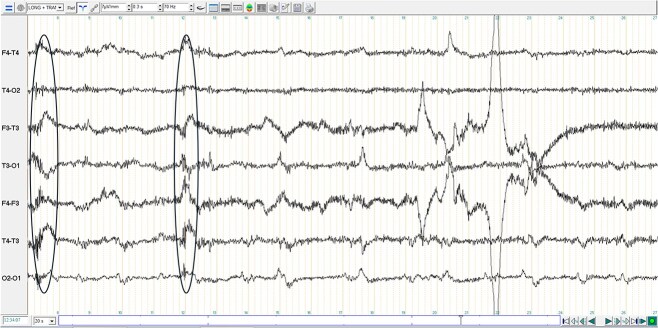
Extract of 20 seconds of the electroencephalographic examination in longitudinal (F2–T4, T4–O2, F3–T3, T3–O1) and transverse (F4–F3, T4–T3, O2–O1) bipolar montage. Artifacts of eye movements visible more pronouncedly on the temporal channels and spreading to the other recording channels. Circled areas highlight some eye movements. Note the artifact changes in F3 (*t* = 7.5 and 12 s), which demonstrates the left lateralization of the eye movements. Although no obvious epileptic activity can be visualized during this recording, this could have been masked by movement artifact.

In the next morning, the dog was premedicated with butorphanol (Butador, Vetviva Richter, Austria; 0.1 mg/kg IV) with a marked sedative effect, induced with propofol (PropoFlo Plus, Zoetis, Belgium; 3 mg/kg IV) and sevoflurane (Sevotek, Animalcare, Spain) used for maintenance under general anesthesia (GA). The head magnetic resonance imaging (MRI) (Magneton Avanto 1.5 Tesla, Siemens) protocol included a dorsal, sagittal and transverse T2-weighted (T2-W), transverse T1-weighted (T1-W) pre- and post-contrast (gadoteric acid, 0.1 mmol/kg intravenously, Dotarem, Guerbet), transverse T2-W fluid attenuated inversion recovery (T2-W FLAIR), T2-W transverse gradient echo (T2-W GRE) and diffusion weighted image (DWI) with apparent diffusion coefficient (ADC) also measured. MRI findings included a unilateral (right-sided) intra-axial multifocal gray matter lesions in the dorsal aspect of the paramedian thalamus, and in all gyri of the temporal, frontal and parietal regions. These lesions were diffusely T2-W hyperintense, T2-W FLAIR hyperintense, T1-W isointense, with “patchy” parenchymal and mild leptomeningeal enhancement (Figure 1). On DWI, the lesions were hyperintense with no abnormal restricted diffusion noted on ADC. The lesions did not demonstrate signal void in T2-W GRE. A suspicion of high intracranial pressure was raised with a moderate right sided midline shift, sulci effacement, mild caudal trans-tentorial herniation and cerebellar coning and mild to moderate mass effect with compression of the right lateral ventricle and third ventricle detected. In the cranial cervical spinal cord, there was mild intramedullary ill-defined T2-W hyperintensity, consistent with an extension of brain disease or syringomyelia secondary to abnormal cerebrospinal fluid (CSF) flow. A continuous rate infusion (CRI) over 20 minutes of mannitol (Mannitol, Frenesius Kabi, UK; 0.5 g/kg IV) and dexamethasone (Colvasone 0.2%, Norbrook, UK; 0.1 mg/kg IV) were given while a lumbar CSF collection over L6-L7 was performed. CSF analysis revealed a total nucleated cell count of 224 cells/μl (reference range 0 to 5/μl), red blood cells 2/μl (reference range 0/μl) and total protein 76 mg/dL (reference range < 40 mg/dl). Cytological examination revealed a marked lymphocytic pleocytosis (94% lymphocytes, 4% monocytes/macrophages, and 2% non-degenerate neutrophils). Lymphocytes were mainly small with just few medium to large cells. No infectious agents or atypical cells were observed. While recovering from GA, an electroencephalogram (EEG) was performed (TruScan EEG, version 7 traveler, Deymed diagnostic). Six needle electrodes were used to record (F3, F4, T3, T4, O1, O2), which is an adaptation from the 8-electrode montage.^1^^,^^2^ The recording lasted 37 minutes. During recording, no additional volatile anesthetic or sedative medications were administered. EEG signals were recorded using the following settings: sensitivity 7 μV/mm, high-pass filter set at 0.3 seconds (corresponding to approximately 0.53 Hz), a low-pass filter at 70 Hz, and a 50 Hz notch filter to attenuate electrical interference. These settings are in line with the minimum recording standards recommended by the International League Against Epilepsy and the International Federation of Clinical Neurophysiology.^3^ The EEG showed little artifact, with occasional discrete muscular activity on the F3-T3 channel. There were recurrent medium-amplitude interictal spikes, with amplitudes ranging from 23 to 78 μV, which appeared generalized in transverse montage and localized in the right hemisphere in longitudinal montage (Figure 2). However, considering that the T3 electrode was persistently and significantly contaminated by EMG artifact, a more generalized electroclinical seizure cannot be excluded. Some brief and subtle episodes of monocular nystagmus of the left eye were perceived during recording. The recorded EEG during eye movements (Figure 3), identifies the asymmetrical manifestation in only the left eye (F3). Although epileptic spikes could not be convincingly detected during eye movements, this could have been masked by movement artifact.

Considering the dog’s young age, multifocal diffuse intra-axial hyperintensities on T2W, marked mononuclear pleocytosis on CSF analysis and low risk of exposure for infectious (urban environment, fully vaccinated with no travel history), a presumptive diagnosis of meningoencephalitis of unknown origin (MUO) was made.^4^^,^^5^ The dog was administered dexamethasone (Colvasone 0.2%, Norbrook, UK; 0.3 mg/kg, IV), cytarabine (Cytarabine injection, Hospira, USA; 200 mg/m_2_, IV) on a CRI over 8 hours and phenobarbital (Phenobarbital Sodium, Martindale Pharma, UK; 4 mg/kg, IV q6h) with close monitoring at the intense care unit. No further motor seizure activity was noticed. Eight hours after recovery from GA, the dog developed acute deterioration in neurological status. This consisted in clinical signs consistent with high intracranial pressure, including comatose state, miotic pupils and bradycardia alongside hypertension (Cushing’s reflex), which led to a cardiorespiratory arrest. The dog died, despite an attempt with cardiorespiratory resuscitation. A post-mortem examination was not conducted in accordance with the owner’s wishes.

## Discussion

Monocular nystagmus is a rare ocular clinical sign where jerk movements occur in only one eye spontaneously. This is reported in human medicine secondary to syphilis, secondary to congenital or acquired ipsilateral vision loss, *spasmus nutans*, multiple sclerosis, alternating hemiplegia of childhood, chiasmal tumors, anisometropia, opacities of the media, strabismic amblyopia, chiasmal tumors, cerebrovascular accident, cerebellar degenerative diseases and epilepsy.^6-16^ In veterinary medicine, monocular nystagmus is scarcely described. Belgian sheepdogs with hereditary chiasmatic malformations (hemi or achiasmatic) can have uniocular saccadic movements.^17^^,^^18^ The optic chiasma of the dog reported was within normal limits. Moreover, a 6-year-old female neutered cocker spaniel is reported to have monocular nystagmus secondary to congenital visual loss since being a puppy.^11^ This eye movement abnormality was characterized by an intermittent, slow, coarse, vertical movements of the eye that was giving the impression that the eye was floating. This “wandering” or “searching” eye was considered most likely to be a Heimann-Bielschowsky phenomenon (HBP).^11^^,^^19^ HBP can be monocular or binocular and it is considered benign.^19^ It is related to long-term complete or partial blindness.^19^ The dog in this case was not blind, indicating that monocular HPB is not a plausible diagnosis.

A case of a 36-year-old man with ictal monocular nystagmus and ictal diplopia is described.^20^ In this case, the monocular nystagmus was the result of seizure activity within the right frontocentral, evaluated by EEG, and caused by a right frontal cortical dysplasia.^20^ As seen in the dog described here a combination of ipsilateral strabismus, contralateral nystagmus and facial spasm were also seen. The EEG pattern of this man resembled our dog (although difficult to interpret given muscle artifact) and it was characterized by eye movement artifact in the left side, which evolved to a seizure pattern mainly affecting the right frontocentral region.^20^ This man was also noticed to have a head turn associated with abnormal eye movements.^20^ The dog was presented with a left head turn (contralateral to the side of the lesion) which was exacerbated with episodes of monocular nystagmus.^21^ In a study where seizures were experimentally induced in cats, a contralateral head turn during the ictal phase was observed.^22^ The origin of the head turn for this dog is difficult to ascertain. Other similar epileptic monocular nystagmus in humans are described.^12^^,^^14^^,^^15^

Vestibular seizure episodes are described in dogs.^23^ Diagnosis was made based on interictal EEG spikes at the level of the fronto-parietal and fronto-temporal areas, and positive responsiveness to antiepileptic therapy supported the hypothesis of an epileptic origin of vestibular episodes seen in these cases. In this case, the more obvious focal right sided temporal EEG spikes detected and response to anti-seizure medication, makes an epileptic event more likely. Epileptic vestibular seizures, with nystagmus and strabismus are well described in humans.^13^^,^^24-27^

Monocular nystagmus is described also secondary to medial thalamus and dorsal midbrain infarcts in human medicine.^16^ In dogs, paramedian thalamic lesions can also cause vestibular clinical signs, such as contralateral head tilt, medial strabismus (esotropia) and conjugate bilateral nystagmus.^28^^,^^29^ Half of the dogs with paramedian thalamic lesions had an extension of the suspected vascular lesion to the midbrain.^28^ Interestingly, dorsal midbrain lesions are associated with convergence-retraction nystagmus.^30^^,^^31^ This unique nystagmus is reported as bilateral and conjugate. The dog presented in this report represents a unique display of monocular nystagmus. A relation between this monocular nystagmus and the dorsal paramedian lesion also found on the MRI of this dog cannot be completed excluded. Considering the episodic nature of events, the absence of other vestibular clinical signs, the possible lateralized cranial focal seizure observed, the response to anti-seizure medication, the abnormal mentation during the events and the EEG findings, an epileptic origin for this monocular nystagmus is strongly suspected. However, while some brief and sporadic eye monocular nystagmus were observed during EEG recording, an obvious association could not be established. Still, the presence of EEG abnormalities would support an epileptic nature of the monocular nystagmus.

This case report has some limitations. First, we cannot exclude that deviations seen on EEG were cardiac artifact contaminations, given that there was no electrocardiogram (ECG) recorded concurrently. However, in our assessment, the deviations in question do not appear rhythmic and do not resemble typical ECG artifacts as described elsewhere.^32^ Moreover, the most convincing abnormal EEG findings were interictal discharges. Considering that this dog had brain disease causing generalized tonic–clonic seizures, we cannot exclude that EEG abnormalities are simply a manifestation of the encephalopathy and not the true cause of the monocular nystagmus. Scalp EEG is unable to pick up activities from deeper structures, eg, paramedian thalamus. Although obvious cortical epileptiform activity during eye movement could not be convincingly visualized, this does not rule out an epileptic origin. Finally, the diagnosis of MUO of this dog remains presumptive as no infectious disease titers or postmortem for a definitive diagnosis were performed.

Monocular nystagmus appears to be a possible clinical sign seen in dogs with focal motor seizure activity. While the exact pathophysiology remains uncertain, a nullification of the subcortical eye movement control by a focal cerebral cortical epileptic region is suspected. We suggest that when presented with monocular nystagmus episodes, if combined with further seizure activity, focal forebrain pathology could be considered as the main differential diagnosis.

## Supplementary Material

Video_1_aalaf078

Video_2_aalaf078

Video_3_aalaf078

## Off-label antimicrobial declaration

Authors declare no off-label use of antimicrobials.

## Institutional animal care and use committee or other approval declaration

Authors declare no institutional animal care and use committee or other approval was needed.

## Abbreviations

ADC, apparent diffusion coefficient
CRI, continuous rate infusion
CSF, cerebrospinal fluid
DWI, diffusion weighted image
EEG, electroencephalogram
GA, general anesthesia
HBP, Heimann-Bielschowsky phenomenon
MUO, meningoencephalitis of unknown origin
MRI, magnetic resonance imaging
T2W, T2-weighted
T1W, T1-weighted
T2-W FLAIR, T2-W fluid attenuated inversion recovery
T2-W GRE, T2-W transverse gradient echo
